# Nitrogen diagnosis based on dynamic characteristics of rice leaf image

**DOI:** 10.1371/journal.pone.0196298

**Published:** 2018-04-24

**Authors:** Yuanyuan Sun, Shaochun Zhu, Xuan Yang, Melanie Valerie Weston, Ke Wang, Zhangquan Shen, Hongwei Xu, Lisu Chen

**Affiliations:** 1 Institute of Applied Remote Sensing and Information Technology, College of Environmental and Resource Sciences, Zhejiang University, Hangzhou, Zhejiang Province, China; 2 College of Ocean Science and Engineering, Shanghai Maritime University, Shanghai, China; College of Agricultural Sciences, UNITED STATES

## Abstract

Digital image processing is widely used in the non-destructive diagnosis of plant nutrition. Previous plant nitrogen diagnostic studies have mostly focused on characteristics of the rice canopy or leaves at some specific points in time, with the long sampling intervals unable to provide detailed and specific “dynamic features.” According to plant growth mechanisms, the dynamic changing rate in leaf shape and color differ between different nitrogen supplements. Therefore, the objective of this study was to diagnose nitrogen stress levels by analyzing the dynamic characteristics of rice leaves. Scanning technology was implemented to collect rice leaf images every 3 days, with the characteristics of the leaves from different leaf positions extracted utilizing MATLAB. Newly developed shape characteristics such as etiolation area (EA) and etiolation degree (ED), in addition to shape (area, perimeter) and color characteristics (green, normalized red index, etc.), were used to quantify the process of leaf change. These characteristics allowed sensitive indices to be established for further model validation. Our results indicate that the changing rates in dynamic characteristics, in particular the shape characteristics of the first incomplete leaf (FIL) and the characteristics of the 3^rd^ leaf (leaf color and etiolation indices), expressed obvious distinctions among different nitrogen treatments. Consequently, we achieved acceptable diagnostic accuracy (training accuracy 77.3%, validation accuracy 64.4%) by using the FIL at six days after leaf emergence, and the new shape characteristics developed in this article (ED and EA) also showed good performance in nitrogen diagnosis. Based on the aforementioned results, dynamic analysis is valuable not only in further studies but also in practice.

## 1. Introduction

During recent years, spectral analysis and digital image processing technology have been widely used in plant nitrogen (N) diagnosis. This technology has greatly increased the efficiency of monitoring the growth station of field crops in a timely and non-destructive manner, thus providing strong technical support for producing high yields in a favorable environment [1, 2].

Hyperspectral imaging technology is a newly developed technique that combines spectral and spatial information simultaneously. Recent studies have demonstrated the potential of hyperspectral imaging technology for plant nutrition research [3, 4]. However, the high cost of images and the strict requirements of the operating environment have limited its application in practice [5]. Alternatively, digital imagery is commonly used in N diagnosis, which not only records spectral and morphological information but also provides a low-cost alternative with suitable image resolution. Current studies have demonstrated the value of digital images in N diagnosis, in particular for characteristics extracted from digital images providing effective parameters in N diagnosis [6, 7]. Moreover, digital imaging devices are portable and easy to operate for data acquisition, and subsequently allow digital image processing to provide a more practical context in terms of future development [8–10].

Rice plants with N deficiency have some specific symptoms such as small leaves, leaf etiolation from the tip [11–13]. These symptoms are closely related to N content as have been widely shown in previous studies [6, 14]. Statistics show that the sampling interval is commonly around 10 days [7, 10, 15]; however, further studies have shown that some detailed changing processes have been missed within this time period [16]. This suggests that some specific and effective characteristics which appear during a short time period could be missed due to the long sampling interval, and for this reason a short sampling interval is considered important in identifying distinctive “dynamic features” in N diagnosis. Supported by previous studies, rice leaf images contain more specific information than that of canopy images, and they are deemed more suitable for N diagnosis compared to those of the canopy [15, 17, 18]. It is also important to note that N content strongly influences the changing rates in rice leaf characteristics, and it is responsible for their final condition. This indicates the possibility of “dynamic features” having a close relationship with leaf N content, and more importantly identifying a “hidden trait” within rice leaves allowing for a deeper understanding of growth and development [16]. For example, leaf etiolation is a specific symptom of N stress and the etiolation rate can effectively show leaf N stress levels. Therefore, analyzing the temporal and spatial changes in a rice leaf by monitoring leaf growth continuously is meaningful in research and practice.

Dynamic analysis is widely used in plant growth modeling and plant genotypic studies [16, 19, 20]. Duan [21] quantified canopy structure to characterize early plant vigor in wheat genotypes by analyzing a time series of images. Neilson [16] described the growth models of two sorghum hybrids under N and water deficiency by continuous monitoring. Similarly, Poiré [19] combined time series images with destructive harvest of shoots and roots to evaluate the N and phosphorus use efficiency of different genotypic plants. These studies demonstrate a plant’s response to nutrition or water stress from the perspective of plant growth modeling or plant genotypic studies. Nevertheless, the perspective of plant N diagnosis has not yet been considered, and thus further research identifying specific characteristics and building diagnostic models is required for understanding the important dynamic characteristics of N diagnosis.

Based on the aforementioned, it is valuable and feasible to apply dynamic characteristics in N diagnosis. In this study, we focused on analyzing the dynamic changes in rice leaves for N diagnosis. To the best of our knowledge, this is the first attempt to use dynamic changing rates in rice leaf characteristics in N diagnosis. The objectives of this study were to (i) analyze the relationship between N supplement and the dynamic characteristics of rice leaves, (ii) explore an approach to quantify dynamic characteristics, and (iii) identify effective dynamic characteristics and establish N diagnostic model.

## 2. Materials and methods

### 2.1 Experimental design

Experiments were implemented during both 2014 and 2015, with the application of hydroponics to cultivate rice plants under different levels of N stress. Rice seeds (cultivar *ZheYou-NO*.*1*) were pre-germinated for 3 days, in moist sand at 26°C. After 15 days’ cultivation, seedlings were individually transplanted in 5 L polyvinyl chloride pots that contained precisely controlled nutrient solution. Experiments were conducted in a glasshouse at Zijingang campus, Zhejiang University (30°17’ N, 120°05’ E, Hangzhou, China). Plants were grown under natural light conditions. The temperature of the glasshouse was programmed to be 30°C/25°C (day/night) and the relative humidity 50%. Nutrient solution formula from the International Rice Research Institute (IRRI) was used to cultivate the rice plants and replaced every 15 days. The experiment applied four different N level treatments, with five replications for each level. Four N levels (ammonium nitrate N1: 0 mg/L, N2: 57.20 mg/L, N3: 85.70 mg/L and N4: 114.30 mg/L, respectively) via hydroponic solutions were applied to different pots. N1 represented extreme deficiency, N2 medium deficiency, N3 slight deficiency and N4 normal supplement. Every 5 days the pH of the nutrient solution in each pot was measured and adjusted to 5.5–6.5 using 1 mol/L NaOH.

### 2.2 Image acquisition and processing

For image acquisition, scanning was applied due to its advantages in data acquisition and processing, especially in closed environments that can effectively eliminate the influence of the environment and operator [18, 22]. Rice leaves were scanned (EPSON GT20000, Epson Inc., Suwa, Nagano prefecture, Japan) every 3 days from 20 days after transplanting (DAT 20) to 44 days after transplanting (DAT 44) during both 2014 and 2015. The top four leaves (including the first incomplete leaf) of each rice plant were scanned at the sampling time and experiments were conducted nine times. Then, a total of 1920 rice leaf images were processed in MATLAB 2013b (MathWorks Inc., Natick, Massachusetts, USA). MATLAB provides programs that have been designed to extract leaf color and shape characteristics to describe the dynamic change of leaf extension and etiolation.

### 2.3 Characteristics extraction

#### 2.3.1 Characteristics of leaf shape

Leaf area (LA) and leaf perimeter (LP) were chosen to describe the process of leaf shape change. These parameters were chosen because N deficiency results in stunted growth and therefore the process of leaf extension and etiolation would be distinct among N supplements. Hence, the changing rate in leaf area and perimeter are important characteristics for evaluation of leaf growth status.

**Fig 1:**
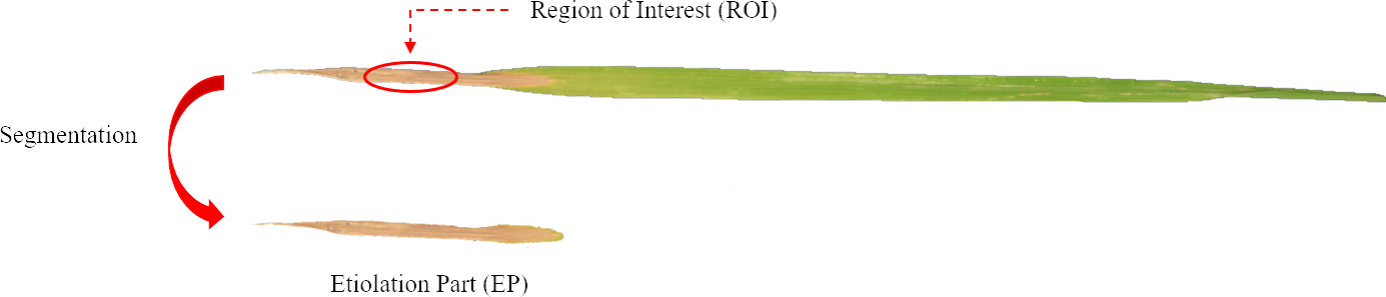
Segmentation of etiolated leaf tip. Etiolation area (EA) and degree of leaf etiolation (ED) were extracted from the 3^rd^ leaf.

Under N deficiency, the N element would be transferred from an old leaf to a young leaf, which would accelerate the process of leaf etiolation in the old leaf. Therefore, the etiolation area (EA) and the degree of leaf etiolation (ED) can be good indicators of N stress levels. Image segmentation was used to extract the etiolated part (Fig 1) and etiolation area was calculated. Based on the aforementioned, EA and ED were calculated to describe leaf etiolation status as follows (Eq (1)):

ED=EA÷LA (1)

#### 2.3.2 Characteristics of leaf color

Color characteristics were extracted from different parts of the leaves according to different leaf positions. Color characteristics of the first incomplete leaf (FIL) were extracted from the entire leaf, because the color of the FIL was generally uniform. However, for a fully expanded leaf, color characteristics were extracted from the leaf tip because this part was more sensitive to N stress than the entire leaf. According to previous research, the leaf tip characteristics performed well in N stress diagnosis [23]. Therefore, rice leaves were trisected along their long axes, and the color characteristics of the first one-third were chosen for further analysis. Color indices (Table 1) examined in previous studies that have good correlations with N content were applied to describe leaf color variations [6, 15, 24].

**Table 1:** Formula and explanation of different characteristics.

Characteristics	Name	Abbrev.	Formula
**Leaf shape**	Leaf area	LA	
	Leaf perimeter	LP	
	Etiolation area	EA	
	Etiolation degree	ED	ED = EA / LA
**Leaf color**	Green	G	
	Normalized red index	NRI	NRI = R/(R+G+B)
	Excess red vegetation index	ExR	ExR = 1.4NRI−NGI
	Excess green vegetation index	ExG	ExG = 2NGI−NRI−NBI
	Dark green color index	DGCI	DGCI = {(Hue−60)/60+(1−Saturation)+(1−Brightness) }/3

### 2.4 Dynamic characteristics analysis

#### 2.4.1 Data processing of dynamic characteristics

1) Growth pattern fitness

To identify the rice plant growth pattern response to N treatments, different types of mathematical models were applied to the leaf shape data such as the power, exponential and logistic functions. The suitability of growth models was evaluated using two statistical methods: coefficient of determination (R^2^) and Akaike information criterion (AIC). R^2^ was used to evaluate the goodness of model fitting and AIC was used to estimate the complexity of model. This process was carried out in IBM SPSS 22.0 (IBM, Armonk, New York, USA).

2) Quantification of dynamic characteristics

Relative growth rate (RGR) is an essential parameter that is widely used in botanical science to evaluate plant biomass change [16, 19]. It was chosen to describe the dynamic changing rate of rice leaf shape under different N stress. RGR (LA, LP, EA and ED) was calculated according to Hunt [25] as follows:

RGR=(ln⁡W2−ln⁡W1)÷(t2−t1) (2)

where W1 and W2 are the initial and final characters (LA, LP, EA and ED) at the beginning (t1) and end (t2) of the measurement period, respectively (Eq (2)).

On the other hand, the average changing rate (ACR) derived from the absolute growth rate (AGR), another important parameter in botanical science, integrates plant color indices with time. It was used to describe the dynamic changing of leaf color. ACR was calculated as follows:

ACR=(X2−X1)÷(t2−t1) (3)

where X1 and X2 are the initial and final characters (G, NRI, DGCI, ExG and ExR) at the beginning (t1) and end (t2) of the measurement period, respectively (Eq (3))

**Fig 2:**
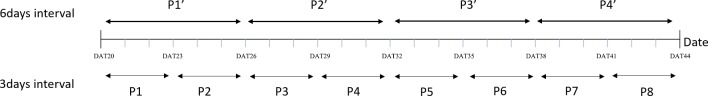
Establishment of data sets. P1 to P8 represent the data set calculated using a 3-day interval. P1’ to P4’ represent a data set calculated using a 6-day interval.

RGR (leaf shape) and ACR (leaf color) were calculated with the time interval between t1 and t2 being set at 3 days or 6 days. However, because of the senescence of old leaves, some data were missing during the late stage. Consequently, in this study, there were 7 data sets with a 3-day interval and 3 data sets with a 6-day interval used for further analysis (Fig 2).

#### 2.4.2 Statistical analysis method

The quantified dynamic characteristics of leaf shape and color were applied in parameter evaluation and model establishment.

(1) Selection of optimal characteristic parameters

One-way analysis of variance (ANOVA) was conducted to estimate parameters and screen optimal parameters for further analysis. F-values and p-values were used to evaluate the inter-group and intra-group differences. A p-value less than 0.05 means the difference is significant and the indices were effective in classification. The optimal parameters to be used in model establishment were selected according to p-value and F-value.

(2) Model establishment and validation

To calculate the optimal diagnostic time and leaf position, a total of 10 sets of data sets (7 using a 3-day interval and 3 using a 6-day interval) from different growth stages were used in model establishment and validation. However, to make a further exploration of dynamic characteristics, we combined the single data sets in time order to improve the diagnostic effect (Table 2). LibSVM was adopted to build the N diagnostic model, and leave one out cross validation (LOOCV) was used to validate the model in MATLAB.

**Table 2:** Combination method of data sets for diagnosis.

Time interval	Single data set	Combined data set
**3days**	P1	——
	P2	P1 P2
	P3	P1 P2 P3
	P4	P1 P2 P3 P4
	P5	P1 P2 P3 P4 P5
	P6	P1 P2 P3 P4 P5 P6
	P7	P1 P2 P3 P4 P5 P6 P7
**6days**	P1’	——
	P2’	P1’ P2’
	P3’	P1’ P2’ P3’

## 3. Results

### 3.1 Dynamic change analysis

#### 3.1.1 Dynamic change of leaf expansion

Model fitting results of the power, exponential and sigmoidal logistic functions are shown in Table 3. All three models produced a curve with an R^2^ greater than 0.80. In particular, the sigmoidal logistic function produced a curve an R^2^ near 0.99 which was higher than that of the others. In contrast to the R^2^, a lower AIC value represents a higher quality model. The AIC of the sigmoidal logistic function ranged from -17.3333 to 1.2889 in the leaf area growth model, and from 4.3837 to 14.5591 in the leaf perimeter model, both of which were obviously less than those of the other models. Utilizing the aforementioned information, the sigmoidal logistic function was chosen for subsequent analysis.

**Table 3:** Growth model fitted leaf area and perimeter data from of N treatments with and R^2^ and AIC values.

Leaf character	Model	Parameter	N1	N2	N3	N4
**Leaf area**	Power	AIC	12.72	20.5153	30.0601	29.9454
		R^2^	0.8032	0.8650	0.8397	0.8636
	Exponential	AIC	-5.277	-0.2981	9.6517	16.0086
		R^**2**^	0.9106	0.9525	0.9415	0.9103
	Sigmoidal logistic	AIC	-17.3333	-15.5123	1.2889	1.085
		R^2^	0.9888	0.9955	0.9897	0.9909
**Leaf perimeter** ** **	Power	AIC	33.6971	38.5181	36.1168	45.2415
		R^2^	0.9252	0.9292	0.9565	0.9143
	Exponential	AIC	28.5178	21.7473	20.4250	22.4331
		R^2^	0.9879	0.9866	0.9878	0.9877
	Sigmoidal logistic	AIC	4.3837	10.8562	12.8223	14.5591
		R^2^	0.9992	0.9875	0.9909	0.9906

**Fig 3:**
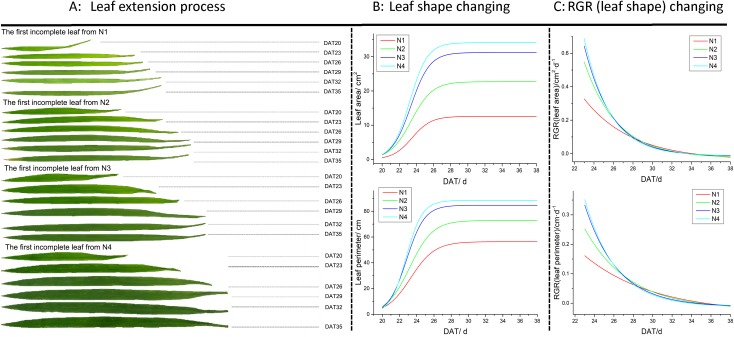
Dynamic changes of the first incomplete leaf in different N treatments.

The extension process of leaves from emergence to full expansion containing different N supplements are shown in Fig 3. Our results indicate that the N supplement influences the color and size of rice leaves, with rice leaves becoming greener and larger with a higher N supplement (Fig 3A), and the growth rate of leaf area and leaf perimeter increased with the increasing N supply (Fig 3B). Overall, it took approximately 10days to reach the peak of leaf area (perimeter). Furthermore, different variation among the four treatments could also be observed in the RGR changes. As shown in Fig 3C, RGR decreased with leaf extension and the leaves with higher N supply initially showed greater RGR (leaf area and perimeter) values. Consequently, bigger decline in RGR value could be observed in higher N treatment.

#### 3.1.2 Dynamic change in leaf color during etiolation

**Fig 4:**
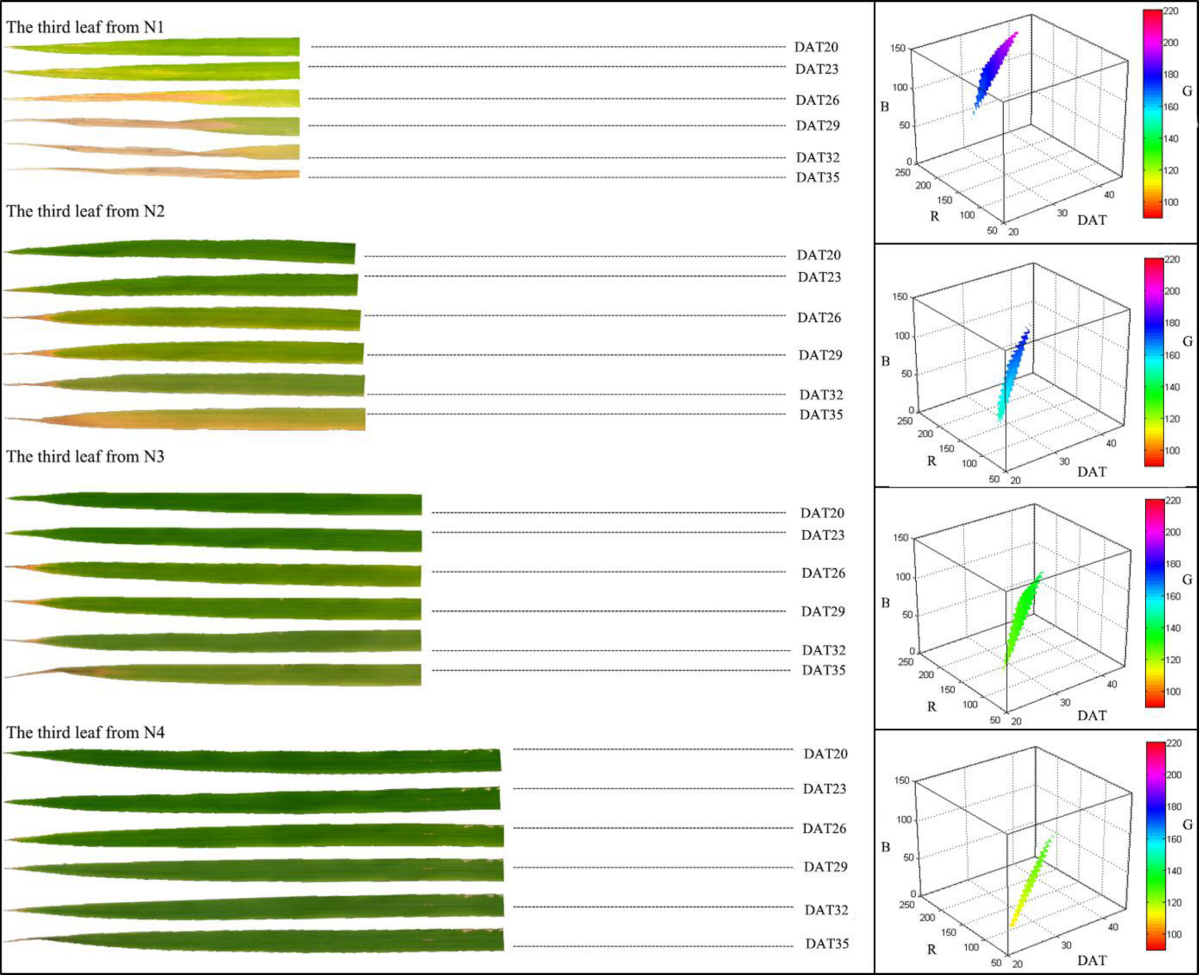
Leaf etiolation process under different N treatments. “X axis” represents time (DAT: day after transplanting), “Y axis” represents red (R), the “Z axis” represents blue (B) and the color bar represents green (G).

The etiolation process of the leaf tip showed that the leaves became “yellower” and “narrower” with decreasing N supply, and the etiolation area increased more rapidly under low N treatments compared to that of high N treatments (Fig 4). The dynamic changing of leaf color was observed utilizing the “slice function” in MATLAB which plotted the relationship between RGB value and time (DAT). This showed that leaves with higher N content produced a lower RGB value. During leaf etiolation, the RGB value increased at different rates and the increasing RGB value meant leaves were losing their “green” and becoming “yellower”

### 3.2 Optimal characteristic parameters in discrimination

Leaf samples were divided into four groups according to leaf position: the first incomplete leaf (FIL), and the 1^st^, 2^nd^, and 3^rd^ fully expanded leaves from the top of the rice plant. Table 4 shows the F value ranged from 0.04 to 44.67. Characteristic parameters whose F values were greater than 2.92 (the p values were less than 0.05) had an obvious difference among the different N treatments.

**Table 4:** F value of rice leaf characteristic parameters using ANOVA.

**3days interval**											
**Data set**	**Index**	**FIL**	**1**^**st**^ **leaf**	**2**^**nd**^ **leaf**	**3**^**rd**^ **leaf**	**Data set**	**Index**	**FIL**	**1**^**st**^ **leaf**	**2**^**nd**^ **leaf**	**3**^**rd**^ **leaf**
P1	LP	18.47^c^	2.51	0.09	1.03	P4	G	2.28	3.49^a^	2.38	0.89
	LA	12.41^c^	1.3	0.48	1.13		NRI	1.43	1.14	3.59^a^	3.83^a^
	EA	——	——	——	2.36		ExR	1.97	1.17	1.9	2.79
	ED	——	——	——	7.93^c^		ExG	2.26	2.02	2.38	1.14
	DGCI	9.04^c^	0.34	2.7	1.78	P5	LP	0.97	1.25	1.78	3.03^a^
	G	1.82	1.39	1.96	0.24		LA	1.22	0.68	3.21^a^	2.09
	NRI	8.11^c^	0.35	1.04	0.42		EA	——	——	——	2.07
	ExR	3.58^a^	1.12	1.7	0.40		ED	——	——	——	4.78^b^
	ExG	1.15	1.39	1.2	0.93		DGCI	0.26	0.82	0.07	1.87
P2	LP	10.16^c^	0.4	1.03	0.18		G	1.17	0.71	1.24	44.67^c^
	LA	7.15^c^	0.9	0.49	1.15		NRI	1.27	0.23	2.7	14.89^c^
	EA	——	——	——	1.31		ExR	1.58	1.62	1.18	38.60^c^
	ED	——	——	——	0.73		ExG	1.64	0.62	0.39	37.82^c^
	DGCI	0.51	0.5	1.42	2.92^a^	P6	LP	1.75	0.53	0.29	0.18
	G	3.36^a^	1.05	0.8	1.41		LA	1.1	0.26	0.5	0.20
	NRI	0.62	1.96	0.8	3.85^a^		EA	——	——	——	1.76
	ExR	4.13^a^	3.03^a^	1.71	2.02		ED	——	——	——	1.07
	ExG	4.72^b^	1.05	0.8	1.25		DGCI	0.1	5.78^b^	5.43^b^	4.85^b^
P3	LP	2.93^a^	1.13	0.94	1.28		G	2.32	1.33	5.54^b^	0.11
	LA	3.15^a^	1.95	1.11	2.40		NRI	3.31^a^	6.20^b^	12.40^c^	0.54
	EA	——	——	——	4.22^a^		ExR	0.65	1.27	16.99^c^	0.10
	ED	——	——	——	16.58^c^		ExG	3.20^a^	7.43^c^	7.54^c^	0.06
	DGCI	3.81^a^	5.97^b^	1.84	1.55	P7	LP	1.2	0.93	0.94	1.25
	G	0.5	2.01	3.93^a^	16.21^c^		LA	0.89	0.6	0.87	0.19
	NRI	1.35	1.59	3.29^a^	6.78^c^		EA	——	——	——	1.02
	ExR	0.73	1.19	1.99	23.11^c^		ED	——	——	——	1.14
	ExG	0.5	0.34	1.38	16.10^c^		DGCI	0.6	0.26	0.11	3.01^a^
P4	LP	1.07	1.4	0.9	0.80		G	2.4	7.34^c^	11.38^c^	0.21
	LA	0.6	0.19	0.40	1.35		NRI	1.1	6.40^c^	1.79	0.16
	EA	——	——	——	2.10		ExR	1.52	1.33	2.93^a^	0.35
	ED	——	——	——	0.71		ExG	0.72	2.78	8.88^c^	0.04
	DGCI	2.73	1.4	0.24	1.36	——	——	——	——	——	——
**6days interval**											
**Data set**	**Index**	**FIL**	**1**^**st**^ **leaf**	**2**^**nd**^ **leaf**	**3**^**rd**^ **leaf**	**Data set**	**Index**	**FIL**	**1**^**st**^ **leaf**	**2**^**nd**^ **leaf**	**3**^**rd**^ **leaf**
P1'	LP	31.41^c^	1.77	0.68	1.23	P2'	G	0.13	5.24^b^	8.19^c^	9.41^c^
	LA	35.44^c^	0.56	0.1	1.66		NRI	5.98^b^	1.58	0.91	7.16^c^
	EA	——	——	——	5.96^b^		ExR	1.33	2.89	1.79	9.71^c^
	ED	——	——	——	6.84^b^		ExG	0.13	0.99	2.03	4.70^a^
	DGCI	7.27^c^	1.23	1.76	2.21	P3'	LP	2.41	0.78	2.64	0.3
	G	3.73^a^	0.89	1.15	0.88		LA	0.70	0.56	0.35	0.7
	NRI	5.00^b^	1.7	3.82^a^	2.7		EA	——	——	——	0.66
	ExR	3.26^a^	0.58	2.27	2.9		ED	——	——	——	5.07^a^
	ExG	3.73^a^	1.67	0.98	0.89		DGCI	0.66	2.02	4.35^a^	1.28
P2'	LP	0.2	2.04	5.36	2.75		G	1.13	0.97	4.82^b^	7.91^b^
	LA	0.53	0.33	1.52	3.45^a^		NRI	0.91	2.69	8.18^c^	3.58^a^
	EA	——	——	——	1.65		ExR	1.01	0.76	4.82^b^	5.34^a^
	ED	——	——	——	2.14		ExG	1.13	4.99^b^	8.19^c^	7.96^b^
	DGCI	0.96	2.19	1.36	5.08^b^	——	——	——	——	——	——

“a” represents *p*-value <0.05

“b” represents *p*-value < 0.01

“c” represents *p*-value < 0.001.

From the distribution of parameters whose p-values were less than 0.05, most were extracted from the 3^rd^ leaf, followed by the FIL, 2^nd^ leaf and 1^st^ leaf. It was observed that leaves from different leaf positions had different growth statuses; in fully expanded leaves (which includes the 1^st^, 2^nd^ and 3^rd^ leaves), dynamic changes in color characteristics were more sensitive to N stress than shape characteristics during the leaf etiolation stage. For the FIL, it was the shape characteristics that showed more distinction among the N treatments during the leaf extension stage.

The P1, P2, P3 and P1’ data sets for the FIL were established during the leaf extension stage, and their shape indices (LA and LP) had higher F values than those of the color indices. Meanwhile, the P3P4P5 and P2’ data sets of the 3^rd^ leaf were built during the leaf etiolation stage, and the color indices (DGCI, G, ExG, ExR, and NRI) and etiolated indices (ED, EA) performed better than LA and LP. Similarly, the color characteristics of the 1^st^ leaf and 2^nd^ leaf in the P6, P7 and P3’ data sets had a higher F value.

### 3.3 Model calibration and validation

#### 3.3.1 Selection of optimal leaf position

As indicated in Table 2, 18 data sets were established in every leaf position. Every data set was used to establish a diagnostic model. Table 5 showed the leaf position that achieved the best validation accuracy for each data set. Overall, the FIL and the 3^rd^ leaf performed better than the other leaves, and the combined data sets achieved higher accuracy than single data sets.

**Table 5:** Leaf position which got the best diagnostic accuracy in every data set.

Time interval	Data set	Leaf position	Training (%)	Validation (%)
**3days**	P1	FIL	63.6	54.5
	P2	FIL	68.2	55.5
	P3	3^rd^ leaf	69.7	57.6
	P4	1st leaf	63.4	58.5
	P5	3^rd^ leaf	60.6	48.5
	P6	FIL	56.8	40.9
	P7	1st leaf	52.2	47.8
**6days**	P1’	FIL	77.3	64.4
	P2’	3^rd^ leaf	62.8	55.8
	P3’	3^rd^ leaf	64.7	61.8
**3days combined data sets**	P1.P2	FIL	74.4	62.8
	P1.P2.P3	3^rd^ leaf	70.9	57.6
	P1.P2.P3.P4	3^rd^ leaf	69.7	63.6
	P1.P2.P3.P4.P5	3^rd^ leaf	69.5	59.1
	P1.P2.P3.P4.P5.P6	2^nd^ leaf	78.0	56.1
	P1.P2.P3.P4.P5.P6.P7	FIL	80.5	72.7
**6days combined data sets**	P1’.P2’	3^rd^ leaf	71.2	68.6
	P1’.P2’.P3’	3^rd^ leaf	67.6	64.7

In single data sets, “dynamic features” of P1, P2 and P1’ were more distinctive in FIL than in other leaves as the FIL was captured during the extension stage, P1’ from the FIL got the best accuracy among them (training accuracy 77.3%, validation accuracy 64.4%). For the P3, P5, P2’ and P3’ data sets, the 3^rd^ leaf showed obvious etiolation, dynamic characteristics of the 3^rd^ leaf were more sensitive than those of the others. During the late stage (P6 and P7), the upper leaves (FIL and 1^st^ leaf) performed better than the lower leaves (the 2^nd^ leaf and 3^rd^ leaf). In combined data sets, the FIL did best in the combined P1P2 and P1-P7 data sets, and P1-P7 achieved the highest accuracy (training accuracy 80.5%, validation accuracy 72.7%). For the combinations that contained P3-P6 (or P2’-P3’) data sets, the older leaves performed better than the others.

#### 3.3.2 Selection of best diagnostic time

Since the FIL and the 3^rd^ leaf performed better than the others in N diagnosis, we further combined these two leaves in the model establishment to improve diagnostic accuracy (Table 6).

**Table 6:** Diagnostic accuracy of combining different leaf positions.

Time interval	Single data set	Training (%)	Validation (%)	Combined data set	Training (%)	Validation (%)
**3days**	P1	72.7	59.1	P1.P2	81.8	65.9
	P2	70.5	54.5	P1.P2.P3	84.1	72.7
	P3	72.7	52.3	P1.P2.P3.P4	63.6	61.4
	P4	61.4	34.1	P1.P2.P3.P4.P5	52.3	43.5
	P5	56.8	45.9	P1.P2.P3.P4.P5.P6	45.5	59.1
	P6	25.0	45.5	P1.P2.P3.P4.P5.P6.P7	45.5	47.7
	P7	54.5	31.8	——	——	——
**6days**	P1’	76.7	60.5	P1’.P2’	74.1	62.8
	P2’	62.8	67.4	P1’.P2’.P3’	55.8	81.4
	P3’	65.1	60.5	——	——	——

In single data sets, the diagnostic accuracy (both 3-days interval and 6-days interval) decreased with time and performed poorly. The best accuracy appeared in the P2’ data set, because the optimal characteristics of the FIL and 3^rd^ leaf were in different data sets, the accuracy did not improve significantly. Subsequently, we combined optimal data sets for further analysis. Data sets P1P2P3 achieved the optimal accuracy (training accuracy 84.1%, validation accuracy 72.7%), but the performance of the other combinations was poor.

## 4. Discussion

Our results show that although data acquisition in dynamic analysis is more time-consuming compared to that of other methods, it still has several distinct advantages that allow effective characteristics to be identified in nitrogen diagnosis.

### 4.1 Importance of “dynamic features” in N diagnosis

(1) In plant N deficiency studies, the sampling interval was too long to detect detailed “dynamic features”, and some specific characteristics were missed [26, 27]. According to plant growth mechanisms, plant responses to different N stress tend to be dynamic and biomass accumulation rate varies with N content. Consequently, a short sampling time is necessary to identify specific “dynamic features” in N diagnosis.
(2) Time series analysis of plant images using non-destructive technology provides both quantitative and qualitative information to understand physiological phenomena such as plant growth pattern responses to environmental change [28, 29]. The results here are in agreement with previous studies and this consequently could be applied in plant growth studies [16].
(3) Dynamic analysis is important for plant studies to identify “hidden traits,” particularly in terms of nutrition diagnosis [16]. In this study the dynamic change in leaf etiolation was quantified using EA and ED, with the results (Table 4) indicating that ED and EA achieved good performance in dynamic analysis.
(4) As is known, “dynamic features” exist over the entire life of a rice leaf; this indicates that N diagnosis can be implemented earlier. According to Tables 5 and 6, using the FIL can achieve an acceptable validation accuracy of 64.4% at DAT 26, and another acceptable validation accuracy of 72.7% calculated at DAT29 was achieved integrating FIL and the 3^rd^ leaf. This is earlier when compared to commonly used methods, implying that diagnosis could be implemented at the emergence of other leaves which would be more meaningful in practices.
(5) To some extent, dynamic analysis of specific characteristics is simple and feasible. Our results show that the dynamic characteristics of the 3^rd^ leaf tip performed well in both ANOVA and modeling. Indicating that using leaf tip information from an old leaf can significantly improve diagnostic operability and efficiency.
(6) Aside from the utilization of N stress diagnosis, dynamic analysis also provides a novel method to identify different types of nutrition deficiency, as indicated in Figs 3 and 4 where leaf extension rate and etiolation rate changed with N supplement. According to plant growth mechanism, nitrogen, phosphorus and potassium deficiency always result in stunted growth, but it differs in terms of new leaf growth rate and old leaf senescence rate. Based on the aforementioned, dynamic characteristics would be distinctive under nitrogen, phosphorus and potassium stress and could be ideal indices for diagnosis.

### 4.2 Application of “dynamic features” in N diagnosis

Leaf shape and spectral information acquired at some particular points in time have been widely used in previous studies, where results have shown that the 3^rd^ leaf or 4^th^ fully expanded leaf could be an ideal indicator in N diagnosis [12, 30, 31]. Our findings (Figs 3 and 4) showed the FIL and 3^rd^ leaf were ideal indicators in dynamic analysis as dynamic characteristics were mainly reflected during the leaf extension and etiolation stages. Dynamic characteristics of the FIL (area, perimeter) and the 3^rd^ leaf (ED, G, ExR, and ExG) showed obvious differences among the N treatments during different growth stages (Table 4). Employing RGR and ACR to quantify the dynamic characteristics, provided an in-depth understanding of the dynamic process of leaf extension and etiolation in regard to N supplement.

It is known to all that knowing plant nutrition status timely and precisely is beneficial to high crop yield and quality. In this study, the N status has been effectively identified by dynamic leaf characteristics which provides valuable information for field management. With the popularization of digital camera, portable scanner and smartphone, farmers can obtain plant dynamic information easily by using these devices. Based on this, the plant nutrition status could be identified by combining with the diagnostic model. Moreover, to determine the optimal fertilization, soil fertility is also needed in quantification of N supplement. By considering the plant nutrition status and soil fertility status, the farmer could adjust the fertilization properly and timely which would be a further application of leaf dynamics.

### 4.3 Effectiveness of N diagnosis using “dynamic features”

In this article, dynamic nature of rice leaf is quantified by different time intervals. As we can see, dynamic characteristics quantified using a 3-days interval and a 6-days interval have their respective advantages in diagnosis. The shorter time interval contributes to early diagnosis and the identification of “hidden traits” which exist in short time, while the longer time interval could help us to further improve the diagnosis accuracy to some extent.

Estimates showed that previous studies have typically collected data near DAT40 when N stress symptoms became notable [15, 32, 33]. Therefore, utilizing “dynamic features” to discriminate N stress levels, our methodology greatly increased the possibility of identifying N stress levels during an earlier stage when stress symptoms were not significant. Exploiting “dynamic features” allowed achievement of a validation accuracy of 64.4% (training accuracy 77.3%) in the FIL at DAT 26, while the 3^rd^ leaf produced higher accuracy at later stage. Because the FIL and the 3^rd^ leaf were the optimal leaves, integrating these two leaves effectively improved the diagnostic accuracy, especially during the early stage data sets P1P2 and P1P2P3 (Table 6). Although the diagnostic accuracy is not as high as that of previous researches, it shows the feasibility of using dynamic characteristics in early diagnosis. In our opinion, sacrificing a certain extent of accuracy for earlier diagnosis is more valuable.

### 4.4 Future work

According to plant growth mechanisms, plant responses to N deficiency embody not only in leaf characteristics, but also in plant physiology and biochemistry. In this paper, we mainly focused on the responses of leaf characteristics to N deficiency. From the perspective of plant physiology and biochemistry, the adaption to N deficiency in metabolism usually results in etiolation and stunted growth. During this process, the expression of N metabolism related genes in the shoots (nitrate reductase (NR1), nitrate reductase (NR2), glutamine synthetase (GS2), ferredoxin-dependent glutamate synthase (Fd-GOGAT), glutamate dehydrogenase (GDH2), glutamate dehydrogenase (GDH3), etc.) are upregulated under short-term N deficiency. In contrast, their expression would decrease under long-term N deficiency [34–36]. In this sense, the responses of the N metabolism related genes to N deficiency differ with N content. Therefore, exploring the relationship between gene expression, leaf characteristics and N content would contribute to the exploration of effective traits for N diagnosis, thus improving the diagnostic effect.

## 5. Conclusion

This study provided a detailed and feasible research method of using dynamic leaf characteristics in rice N diagnosis. We demonstrated the effectiveness and feasibility of dynamic analysis, in particular for quantifying specific “dynamic features” and allowing for earlier diagnosis of N stress.

The results showed that dynamic characteristics have distinctive differences among N treatments, particularly the dynamic changing of the FIL (area and perimeter) and the 3^rd^ leaf (etiolated indices and color indices) which could be ideal indicators in N diagnosis. In particular, leaf etiolation was quantified using two characteristics developed in this study (EA and ED) and achieved good performance in feature selection.

When applying “dynamic features” in modeling, N stress levels can be discriminated earlier than commonly used methods. Utilizing a single leaf, the FIL performed better than the other leaves during the early stage, achieving an acceptable validation accuracy of 64.4% at DAT26. Furthermore, diagnostic accuracy can be obviously improved during the early stage using combined parameter sets of the FIL and the 3^rd^ leaf.

Finally, this study, from dynamic analysis to diagnostic model establishment, demonstrated the value of “dynamic features” in N diagnosis, thus helping future research both in terms of innovative applications and field management.
